# Bayesian Meta-Analysis of Transcatheter Mitral Valve Edge-to-Edge Repair for Secondary Mitral Regurgitation in Heart Failure

**DOI:** 10.1016/j.jacadv.2026.103078

**Published:** 2026-08-26

**Authors:** James W. Schurr, Tayyab Shah, Ashley Ezema, John Bertot, Amar Rai, Paul Fiorilli, Allison Tsao, Alexander Fanaroff, Jay Giri, Ashwin Nathan

**Affiliations:** Cardiovascular Medicine Division, Perelman School of Medicine, Hospital of the University of Pennsylvania, Philadelphia, Pennsylvania, USA

**Keywords:** Bayesian, heart failure, mitraclip, mitral valve, M-TEER

Transcatheter mitral valve edge-to-edge repair (M-TEER) is a minimally invasive method for reapproximating mitral leaflets in secondary mitral regurgitation (MR) resulting from displaced papillary muscles in dilated cardiomyopathies. Randomized clinical trials have shown conflicting outcomes resulting in meta-analyses to understand pooled treatment effects. However, frequentist methodological limitations led authors to conclude there was “no significant reduction in all-cause or cardiovascular (CV) mortality” in the most recent meta-analysis, despite large point estimates of treatment effects (pooled HR of 0.76 and 0.77, respectively).^1^ Given that a *P* value marginally above 0.05 does not provide information about the true probability of treatment benefit with little clinical guidance, we conducted a Bayesian random-effects meta-analysis to reanalyze this finding.

**What is the clinical question being addressed?**

Among patients with secondary mitral regurgitation and heart failure, does mitral valve edge-to-edge repair reduce mortality and heart failure hospitalization?

**What is the main finding?**

Bayesian meta-analysis of 3 randomized clinical trials showed a 93% probability of all-cause mortality benefit and 96% probability of heart failure hospitalization reduction with mitral valve edge-to-edge repair.

Our review was registered on PROSPERO (https://www.crd.york.ac.uk/PROSPERO/view/CRD420261301624) and performed in accordance with PRISMA guidelines. This study was exempt from Institutional Review Board approval. A systematic search was conducted (February 21, 2026) using PubMed/MEDLINE and ClinicalTrials.gov for randomized clinical trials enrolling adults with heart failure (HF) and at least moderate MR, comparing currently approved M-TEER devices plus guideline-directed medical therapy vs guideline-directed medical therapy alone (full search strategy available in protocol). Trials of primary MR, nonapproved devices, or highly-specific populations were excluded. Study-level data were independently extracted from primary publications, using the longest available follow-up data for each trial.

We analyzed 3 endpoints: 1) all-cause mortality; 2) CV mortality; and 3) HF hospitalizations (HFHs); each using 2 complementary methods of HR pooling (primary) and logistic regression (secondary). To assess prior assumption sensitivity, we specified 5 prior distributions. The primary prior distribution was weakly informative and centered at the null (placing approximately 95% of prior mass between OR 0.37 and 2.72); for sensitivity, we also included vague and skeptical priors. Two additional reference priors were informed by external evidence: the frequentist-informed prior was centered on the pooled HR from the frequentist meta-analysis by Anker et al and the enthusiastic prior was centered on the COAPT 24-month all-cause mortality HR. HR pooling using each trial’s longest available follow-up was the primary analysis, leveraging full time-to-event data and preserving each trial’s prespecified analytic design; logistic regression on binary 24-month outcomes was a sensitivity analysis to harmonize follow-up across trials. Concordance between approaches across all endpoints supports robustness. A Bayesian posterior probability of benefit represents the probability, given the observed data and prior assumptions, that the true treatment effect favors M-TEER (ie, HR or OR <1.0); it is distinct from a frequentist *P* value and directly quantifies the likelihood of a beneficial direction of effect. For all models, between-study heterogeneity was parameterized through a prior on tau (the between-study standard deviation on the log scale). The primary prior specification and 4 of the 5 sensitivity priors (weakly informative, skeptical, frequentist-informed, and enthusiastic) used a half-normal prior (normal[0, 0.3] truncated at zero) for tau; the Vague specification used a heavier-tailed half-Cauchy prior (Cauchy[0, 0.5] truncated at zero), representing a prespecified sensitivity to heterogeneity assumption. All statistical analyses were performed using R (version 4.3.1; R Foundation for Statistical Computing) fitting models with Hamiltonian Markov Chain Monte Carlo sampling.^2^

Three studies met the inclusion criteria: MITRA-FR 2-year follow-up study (n = 304), COAPT 5-year follow-up study (n = 614), and RESHAPE-HF2 (n = 505), for a total enrollment of 1,423 patients.^3^, ^4^, ^5^

Using the primary (weakly informative) prior, M-TEER was associated with a 93% probability of benefit for all-cause mortality, 88% for CV mortality, and 96% for HFH (HR pooling); corresponding estimates using logistic regression were 92%, 82%, and 99%, respectively. These findings were robust across all 5 prior specifications, with probabilities of benefit ranging from 90% to 98% for all-cause mortality, 79% to 97% for CV mortality, and 92% to 100% for HFH (Figure 1).

**Figure 1:**
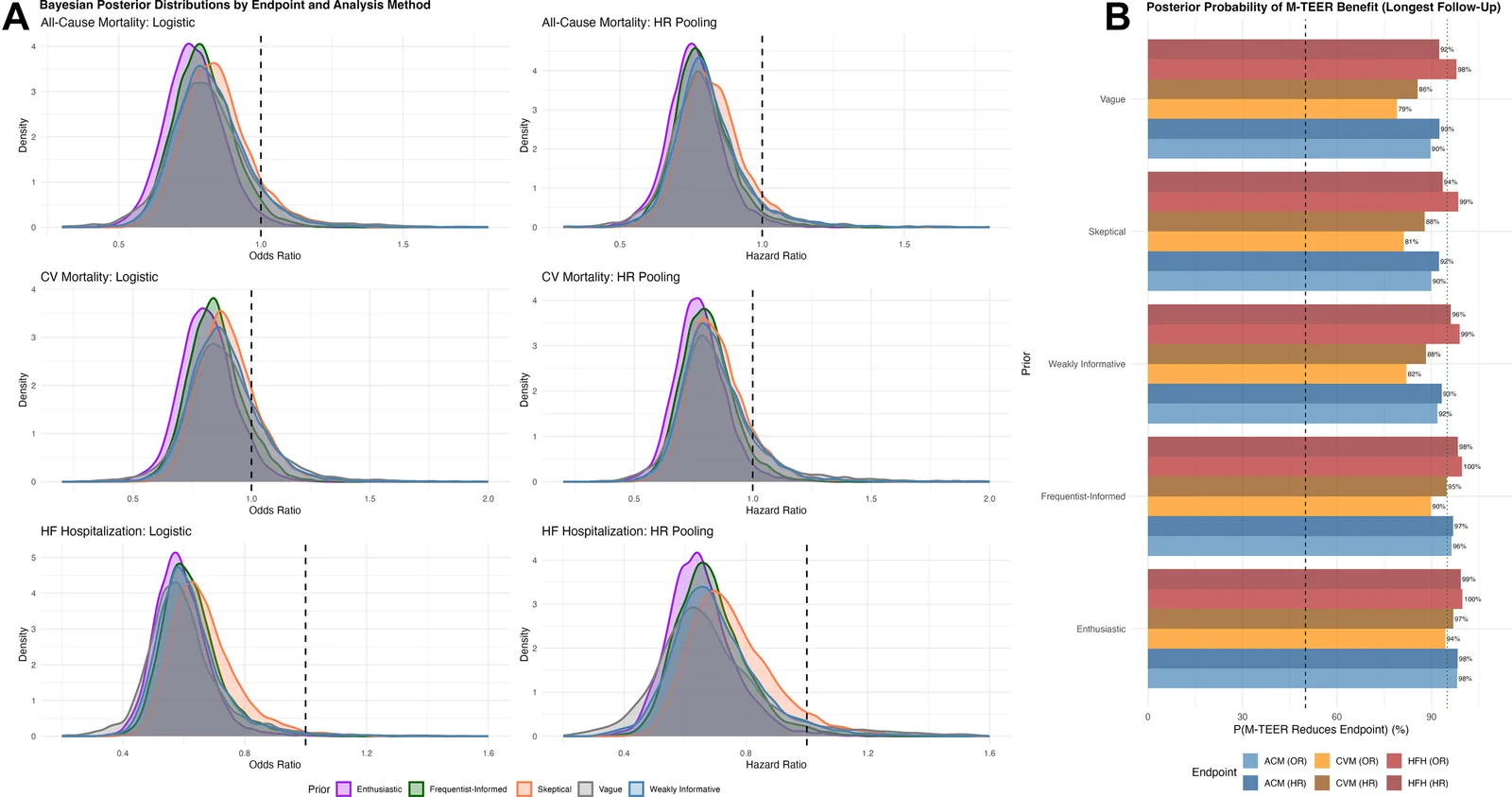
Bayesian Posterior Probability of Benefit by Endpoint and Prior (A) Bayesian posterior distribution by endpoint and analysis method. (B) Posterior probability of M-TEER benefit (longest follow-up). Posterior probability of treatment benefit with M-TEER vs GDMT alone for all-cause mortality, cardiovascular mortality, and heart failure hospitalization, shown across 5 prior distributions (weakly informative, vague, skeptical, frequentist-informed, and enthusiastic). Each panel displays the posterior probability of benefit (HR or OR <1.0) derived from HR pooling and logistic regression models. Probabilities exceeding 90% are considered clinically meaningful. Results were robust across all prior specifications. ACM = all-cause mortality; CV = cardiovascular; CVM = cardiovascular mortality; HF = heart failure; HFH = heart failure hospitalization; M-TEER = transcatheter mitral valve edge-to-edge repair.

Effect estimates were consistent across analytical methods. For all-cause mortality, the pooled OR was 0.81 (95% credible interval [CrI]: 0.61-1.10) and the pooled HR was 0.80 (95% CrI: 0.61-1.14). For CV mortality, OR 0.87 (95% CrI: 0.66-1.20) and HR 0.82 (95% CrI: 0.62-1.16). For HF hospitalization, OR 0.60 (95% CrI: 0.46-0.89) and HR 0.68 (95% CrI: 0.46-1.05). At clinically meaningful thresholds, the posterior probability (HR pooling) of M-TEER conferring at least a 10% relative reduction (P[HR < 0.90]) was 81% for all-cause mortality, 72% for CV mortality, and 92% for HFH; for a 20% reduction (P[HR < 0.80]), corresponding probabilities were 51%, 42%, and 80%, respectively.

Posterior probabilities of benefit were stable across tau prior specifications: under a half-Cauchy tau prior (Cauchy[0, 0.5], Vague specification), probabilities were 92.6%, 85.6%, and 92.4% for all-cause mortality, CV mortality, and HFH, respectively, compared to 92.6%, 88.2%, and 96.1% under the primary half-normal tau prior.

This meta-analysis demonstrates a reduction in mortality and HFH with M-TEER for MR in HF which was consistent across all priors. Although the signal for reduction in CV mortality was less strong, this finding is limited by only a 12-month follow-up from MITRA-FR. Several methodological limitations warrant consideration. First, this analysis is based on 3 trials enrolling 1,423 patients; although the Bayesian framework makes full use of available evidence, the limited number of trials constrains the precision of effect. Second, follow-up duration varied across trials and endpoints (12-60 months), and MITRA-FR contributed only 12-month data for CV mortality, introducing heterogeneity that the random-effects model accommodates but cannot fully eliminate. Third, posterior probabilities reflect the likelihood that the direction of effect favors M-TEER, not the certainty of a specific effect magnitude.

This study adds clinical context to the frequentist meta-analysis by Anker et al. Although they concluded no mortality benefit based on a *P* value of 0.056, our Bayesian reanalysis yielded a 93% posterior probability that the direction of effect favors M-TEER for all-cause mortality. This finding contextualizes, rather than supersedes, the frequentist result and reflects the probability of a beneficial direction of effect rather than the certainty of a specific effect magnitude. The Bayesian framework used here is particularly well-suited to this question: rather than a binary *P* value, it yields a direct probability that treatment is beneficial, a format that more naturally informs clinical decision-making under uncertainty. Based on these findings, we believe it is appropriate for heart teams to continue offering M-TEER to patients with anatomically favorable secondary MR and HF, with high posterior probability of HFH reduction and clinically meaningful probability of mortality benefit.

## Funding support and author disclosures

Dr Fiorilli has received consulting honoraria from 10.13039/100006520Edwards Lifesciences. Dr Giri has received research funding and served on advisory boards for Abiomed, Edwards, Boston Scientific, and Inari Medical; and holds equity in Endovascular Engineering. Dr Fanaroff is supported by 10.13039/100000050NIH/NHLBI R01HL174691; and has received research support from 10.13039/100008497Boston Scientific. All other authors have reported that they have no relationships relevant to the contents of this paper to disclose.
